# Implantable Cardioverter-Defibrillators in Poland Compared with other European Countries from the Patient’s Perspective: Insights from the EHRA Patient Survey

**DOI:** 10.3390/ijerph20065045

**Published:** 2023-03-13

**Authors:** Łukasz Januszkiewicz, Marcin Grabowski, Michał Mirosław Farkowski, Paweł Życiński, Tomasz Jędrychowski, Mariusz Pytkowski, Julian K. R. Chun, Jose M. Guerra, Giulio Conte, Sérgio Barra, Serge Boveda, Maciej Kempa

**Affiliations:** 1 1st Department of Cardiology, Medical University of Warsaw, 02-097 Warsaw, Poland; 2 II Department of Heart Arrhythmia, National Insitute of Cardiology, 04-628 Warsaw, Poland; 3 1st Department of Cardiology, Medical University of Lodz, 91-347 Lodz, Poland; 4 Department of Cardiology, Voivodship Specialist Hospital, 06-400 Ciechanow, Poland; 5 CCB, Cardiology, Med. Klinik III, Markuskrankenhaus, 60431 Frankfurt, Germany; 6 Department of Cardiology, Hospital de la Santa Creu i Sant Pau, Universitat Autònoma de Barcelona, IIB SANT PAU, CIBERCV, 08025 Barcelona, Spain; 7 Cardiology Department, Fondazione Cardiocentro Ticino, 6900 Lugano, Switzerland; 8 Università della Svizzera Italiana Lugano, 6900 Lugano, Switzerland; 9 Cardiology Department, Hospital da Luz Arrabida, 4400-346 Vila Nova de Gaia, Portugal; 10 Heart Rhythm Department, Clinique Pasteur, 31076 Toulouse, France; 11 Heart Rhythm Management Centre, Universitair Ziekenhuis Brussel-Vrije Universiteit Brussel, 1090 Brussels, Belgium; 12 Paris Cardiovascular Research Center, INSERM Unit 970, 75015 Paris, France; 13 Department of Cardiology and Electrotherapy, Medical University of Gdansk, 80-214 Gdansk, Poland

**Keywords:** quality of life, ventricular arrhythmias, sudden cardiac death

## Abstract

Background: The aim of this study was to compare differences between Polish ICD recipients and ICD recipients from other European countries in terms of quality of life, information provision before ICD implantation, and end-of-life issues. Methods: This is a sub-analysis of the “Living with an ICD” patient survey (25-item questionnaire) organized by the European Heart Rhythm Association between 12 April 2021 and 5 July 2021 in ten European countries. Results: There were 410 (22.7%) patients from Poland and 1399 (77.3%) from other European countries. A total of 51.0% of Polish patients reported improvement in their quality of life compared with 44.3% in other countries (*p* = 0.041). Remote monitoring was three times more often utilized in other countries than in Poland (66.8% vs. 21.0%, *p* < 0.001). While 78.1% of Poles felt well informed before ICD implantation compared with 69.6% of subjects from other countries (*p* = 0.001), they were less familiar with the ICD deactivation process than others (38.9% vs. 52.5%, *p* < 0.001). Conclusions: Despite the less frequent use of remote monitoring and gaps in end-of-life issues, Polish ICD recipients reported more favorable quality of life and a higher level of information received before device placement than patients in other European countries.

## 1. Introduction

Implantable cardioverter-defibrillator (ICD) treatment is a guideline-recommended therapy in subjects at high risk of sudden cardiac death [1]. Despite the high effectiveness of this device in sudden cardiac death prevention in specific populations, ICD carries a risk of complications which may affect patients’ life significantly [2]. ICD recipients are at risk of anxiety, depression, and psychological distress, associated mostly with appropriate or inappropriate shocks [3,4,5]. Furthermore, patients should know how an ICD works to understand why it might be helpful in case of sudden cardiac arrest. Accordingly, the 2022 European Society of Cardiology guidelines for the management of patients with ventricular arrhythmias and the prevention of sudden cardiac death highly support informed discussion with patients about quality-of-life issues, shared decision making and ICD deactivation options [1].

Adherence to guidelines is not, however, equal in Europe, which may be exemplified by different ICD implantation rates in countries [6]. Geographic variations in ICD therapy have been studied mostly in terms of implantation rates [7,8]. Other important aspects of ICD recipient life, i.e., quality of life, complications and end of life issues, are less often examined in terms of geographical differences. In this report, we have evaluated differences between the Polish cohort and other European countries participating in the ‘Living with an ICD’ study supported by the European Heart Rhythm Association (EHRA). 

## 2. Materials and Methods

This is a sub-analysis of the “Living with an ICD” patient survey started and organized by the EHRA Scientific Initiative Committee. The complete methodology has been described previously [9]. In brief, a 25-item survey was created and posted on an electronic platform (Supplementary Material S1). The link was sent to the EHRA Research Network centers, national arrhythmia working groups and patient associations working in each participating country whenever possible. The survey was conducted in EHRA Scientific Initiative Committee countries: in Croatia, France, Germany, Italy, Latvia, United Kingdom, Poland, Portugal, Serbia, and Spain between 12 April 2021 and 5 July 2021. The electronic or paper form of the questionnaire was used by the participants already implanted with an ICD (no restrictions regarding ICD dwell time). The subjects were asked to answer the questionnaire autonomously; however, medical staff assistance was possible if necessary. In the survey, mostly demographic data, basic device data, information provision before ICD implantation and end of life issues were collected. Ethics committee approval was obtained where needed according to the local policy.

### Statistical Analysis

Absolute numbers and percentages were shown for categorical variables, and means (with standard deviations) or medians (with interquartile range) were used for continuous variables. The normal distribution of data was tested using the Kolmogorov–Smirnov and Shapiro–Wilk tests. Distributions of categorical data were examined using the x2 test or Fisher’s exact test, as appropriate. A *p*-value < 0.05 was considered statistically significant. The statistical analysis was performed in the SPSS software, version 23.0 (IBM Corporation, Armonk, NY, USA).

## 3. Results

### 3.1. Patient Population

The study group comprised 410 (22.7%) Polish patients and 1399 (77.3%) from other countries (France—550, Germany—248, Spain—228, Italy—138, Latvia—83, United Kingdom—79, Serbia—50, Croatia—20, Portugal—3). The median from first ICD implantation to survey participation was 5 years (IQR 2-10). The Polish cohort was younger, and there were more women than in other countries (Table 1). The Polish subgroup reported a lower level of education than in other countries (*p* < 0.001) and were less often employed than in other countries (*p* < 0.001). There were no differences regarding the device type implanted. Remote monitoring was, however, three times more frequently utilized in other countries than in the Polish cohort. Moreover, a substantial percentage of Polish patients were not aware of whether their device is controlled remotely or not. There was no difference in the complication rate and median time from the first ICD implantation between analyzed subgroups. 

### 3.2. Quality of Life 

More than half of Polish patients reported improvement in their quality of life compared with 44.3% in other countries (*p* = 0.041). There were no differences between the analyzed groups regarding feelings of safety after ICD implantation, depression, acceptance of ICD limitations and significant lifestyle change after ICD implantation (Table 2).

### 3.3. Information Provided to Patients before Implantation

**Figure 1:**
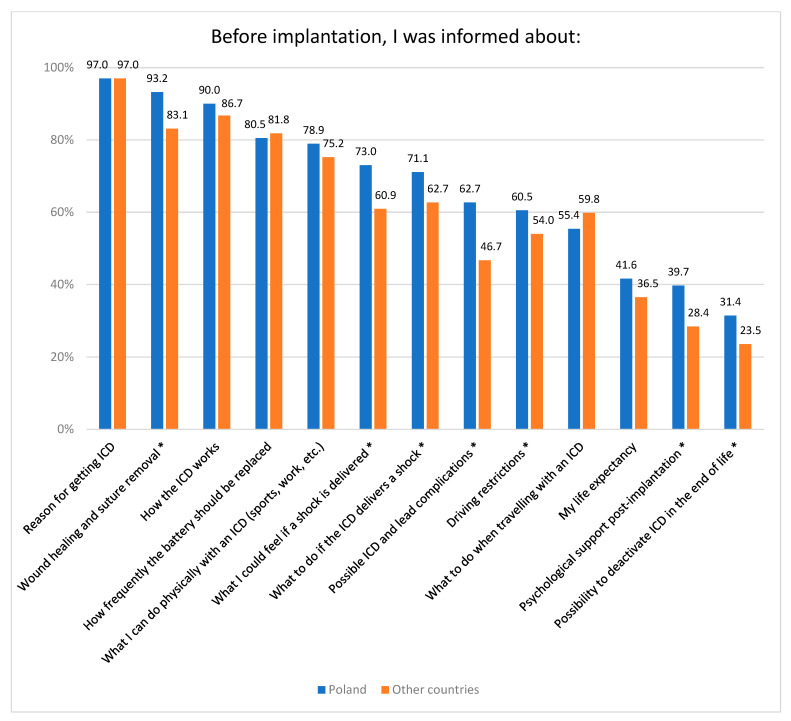
Information provided to patients according to cohorts. * *p* < 0.05.

The Polish cohort more often received a full explanation of available treatment options before ICD implantation, compared with other countries (76.2% vs. 64.2%, *p* < 0.001). The Polish cohort was not as actively involved in the decision-making about the ICD implantation as other countries’ populations (49.5% vs. 54.0%, *p* = 0.022). In general, Polish patients received more complete or similar information before ICD implantation compared with other countries (Figure 1), and almost 80% of Poles felt well-informed before ICD implantation compared with almost 70% of subjects from other countries (*p* = 0.001). 

### 3.4. Patients’ Needs 

**Figure 2:**
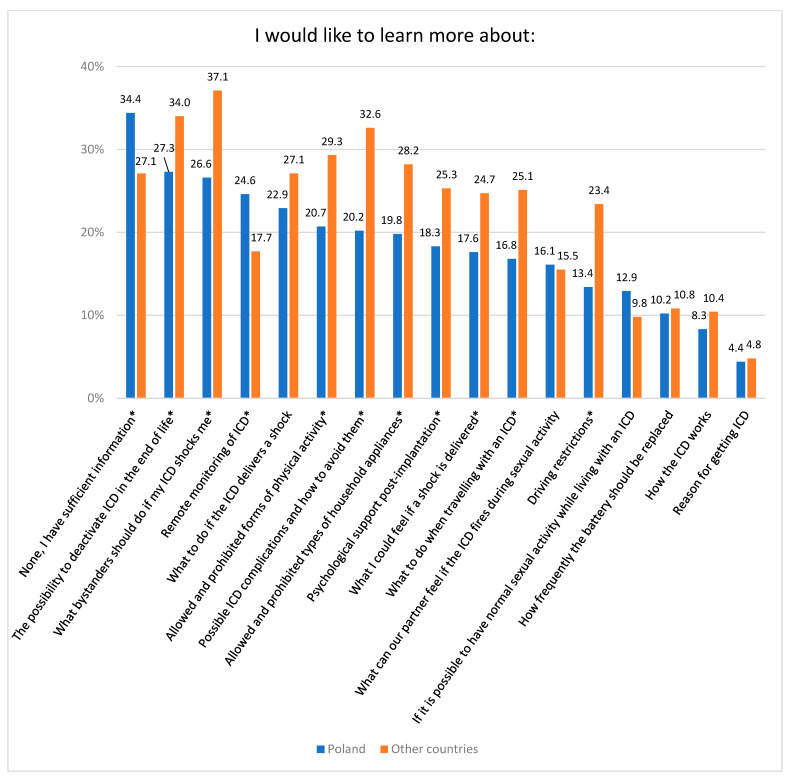
Patients’ needs regarding ICD education according to cohorts. * *p* < 0.05.

In general, the Polish subgroup had minor needs in terms of ICD education compared with other countries, except for remote monitoring. The top three aspects of life that Polish patients would like to learn about were: the possibility of deactivating ICD at the end of life (27.3% compared with 34.0% in other countries, *p* = 0,012), what bystanders should do if my ICD shocks me (26.6% compared with 37.1% in other countries, *p* < 0.001) and remote monitoring (24.6% compared with 17.7% in other countries, *p* = 0.002, Figure 2). 

### 3.5. End of Life 

The Polish cohort reported a lower level of knowledge about end-of-life issues. Before ICD implantation, Poles were more often informed about the possibility of deactivating the ICD at the end of life (31.4% vs. 23.5%, *p* = 0.002), but at the time of filling out the questionnaire fewer Polish patients knew that the ICD may be deactivated at health deterioration (38.9% vs. 52.5%, *p* < 0.001). The Polish cohort less frequently understood why ICD deactivation may be helpful (42.3% vs. 58.4%, *p* < 0.001). The Polish cohort reported, however, a similar need for involvement in ICD deactivation decisions as other countries (90.7% vs. 92.6%, *p* = 0.225).

## 4. Discussion

This sub-analysis of the ‘Living with an ICD’ patients survey shows significant differences between Polish ICD recipients and ICD recipients from other countries in baseline characteristics, quality of life, provision of information prior to ICD implantation, patients’ needs and end-of-life issues. Polish ICD recipients reported an improved quality of life more often than in other countries. These results might be partially explained by varied demographic data between evaluated groups. The Polish cohort was younger, there were more female patients, more married or living with partner subjects and lower education levels. On the other side, implanted device type, complications rate and the median time from ICD implantation to the questionnaire completion were similar in Poland and other countries. One of the factors that could influence the observed differences was better information provision before ICD implantation in the Polish subgroup than in others. We previously showed that this was associated with favorable outcomes [9]. 

A shared decision-making process is advocated in 2022 ESC guidelines, and this is a relevant part of patient-centered care. Patient participation in the decision about ICD implantation is more informed after careful information provision by healthcare professionals. Although Polish patients received a superior or comparable level of information before ICD implantation, there is still room for improvement. ICD deactivation, psychological support and life expectancy are the areas least often discussed in Poland. Additionally, efforts should be made to increase the active involvement of patients in the decision-making; only almost 50% of the Polish cohort was actively involved in ICD implantation decisions, and approximately one of six was not at all involved in that. Since most of the ICDs are implanted in the primary prevention of sudden cardiac death, there is sufficient time to discuss all the aspects of ICD therapy and answer patients’ doubts. Aspects of life with an ICD which were not discussed widely enough before implantation was often desired in patients’ needs, such as the ICD deactivation process and remote monitoring. 

Remote monitoring benefits are well documented in the ICD population [10,11]. Remote device management is recommended in the ESC guidelines, as well as by the Heart Rhythm Society expert consensus, the Polish Cardiac Society, and recent British Heart Rhythm Society guidelines for the follow-up of cardiac implantable electronic devices [1,12,13,14,15]. Furthermore, a remote monitoring system contributes to increasing patients’ feelings of safety [9,16]. Despite that, physicians still struggle to increase the utilization of remote monitoring; the lack of reimbursement represents the most significant barrier to broader implementation, even during the COVID-19 pandemic [17,18,19]. In this analysis, we observed a huge gap in the implementation of remote monitoring; Polish patients were approximately a third as often controlled with remote monitoring compared with other regions. This could be at least partially explained by significant heterogeneity in reimbursement policies across Europe [20]. An analysis of the baseline characteristics revealed that over 50% of study participants were enrolled in France, Germany and Spain, countries with wide reimbursement policies. Contrary to that, there is a lack of remote monitoring reimbursement in Poland, which is one of the primary barriers for its wider implementation [20,21]. Of note, almost a quarter of Polish patients wanted to learn more about the remote monitoring of an ICD. This was the only aspect of life with an ICD in which Polish patients had higher needs in terms of education than other regions. It might be interpreted as a clear message from the patients to reconsider wider access to remote monitoring in Poland. Recently, a decision was made to implement remote monitoring reimbursement in Poland [22]. 

In addition, another relevant aspect of life which Polish patients wished to discuss was the ICD deactivation process at the end of life. Before ICD implantation, approximately 30% of Polish patients were informed about the possibility to deactivate ICD compared with almost a fourth of other countries’ patients. At the time of the survey, the proportion of patients knowing about the possibility of ICD deactivation increased unequally across analyzed regions (the smallest difference was in Poland). All of the aspects regarding awareness about the ICD deactivation procedure indicated that Polish patients were significantly undereducated. The reasons behind variable practices of ICD deactivation across Europe are not well understood. However, we suspect a bigger reluctance to discuss this difficult topic, lower awareness of deactivation importance and/or lower adherence to guidelines in this specific aspect in Poland. Polish patients recognized the ICD deactivation process at the end of life as the most important aspect of life with an ICD they would like to learn more about. The need for advanced care planning discussion is growing in the ICD population [23].

This study has several limitations typical for survey research. First, inclusion bias cannot be excluded because of the observational character and voluntary participation of the subjects. This may result in unbalanced differences in the basal characteristics, i.e., higher S-ICD representation than usually reported in the literature. The second limitation refers to misreporting of data such as ICD indications or complications by patients. However, in the previous manuscript we showed reasonably good consistency in our patient-generated data with ICD registries. Third, the risk of untrue answers might be present, although we believe the participants had no reason to give those. 

## 5. Conclusions

In summary, Polish ICD recipients reported more favorable quality of life than in other countries. The cause of this effect may be related to the demographic differences and better information provision before ICD implantation in Poland than in other countries. Our findings indicate that higher efforts should be made to increase remote monitoring and end-of-life discussions in Poland. 

## Figures and Tables

**Table 1:** Baseline characteristics according to cohorts.

N (%) Or Median, IQR	Whole Cohort 1809 (100)	Poland 410 (22.7)	Other Countries 1399 (77.3)	*p*-Value
Age				<0.001
0-20	7 (0.4)	2 (0.5)	5 (0.4)	
21-40	271 (15.0)	88 (21.5)	183 (13.1)	
41-60	682 (37.7)	128 (31.2)	554 (39.6)	
61-80	784 (43.3)	174 (42.4)	610 (43.6)	
≥81	65 (3.6)	18 (4.4)	47 (3.4)	
Sex (% female)	624 (34.5)	164 (40.0)	460 (32.9)	0.008
Education level				<0.001
Primary school	147 (8.1)	45 (11.0)	102 (7.3)	
Secondary school	608 (33.6)	234 (57.1)	374 (26.7)	
College	381 (21.1)	36 (8.8)	345 (24.7)	
University	673 (37.2)	95 (23.2)	578 (41.3)	
Employment status				<0.001
Student	21 (1.2)	4 (1.0)	17 (1.2)	
Employed	715 (39.5)	150 (36.6)	565 (40.4)	
Not employed	186 (10.3)	44 (10.7)	142 (10.2)	
Retired	887 (49.0)	212 (51.7)	675 (48.2)	
Marital status				0.006
Married or living with a partner	1351 (74.7)	317 (77.3)	1034 (73.9)	
Single	320 (17.7)	51 (12.4)	269 (19.2)	
Widower or widow	87 (4.8)	29 (7.1)	58 (4.1)	
Living at home (as a child)	8 (0.4)	2 (0.5)	6 (0.4)	
Living alone with children	43 (2.4)	11 (2.7)	32 (2.3)	
Time from first ICD implantation	5 (2-10)	5 (2-9)	5 (2-10)	0.620
Device type				0.573
ICD-VR, ICD-DR	829 (45.8)	181 (44.1)	648 (46.3)	
S-ICD	563 (31.1)	124 (30.2)	439 (31.4)	
CRT-D	281 (15.5)	70 (17.1)	211 (15.1)	
Do not know	136 (7.5)	35 (8.5)	101 (7.2)	
Remote monitoring				<0.001
Yes	1021 (56.4)	86 (21.0)	935 (66.8)	
No	675 (37.3)	267 (65.1)	408 (29.2)	
Do not know	113 (6.2)	57 (13.9)	56 (4.0)	
ICD indication				
Post-cardiac arrest (secondary prevention)	583 (32.2)	136 (33.2)	447 (32.0)	0.642
Prevention of sudden death	808 (44.7)	147 (35.9)	661 (47.2)	<0.001
Heart failure symptoms	665 (36.8)	197 (48.0)	468 (33.5)	<0.001
Do not know	54 (3.0)	14 (3.4)	40 (2.9)	0.561
Complications				
None	1404 (77.6)	308 (75.1)	1096 (78.3)	0.169
Inappropriate shocks	209 (11.6)	58 (14.1)	151 (10.8)	0.062
Malfunctioning lead	204 (11.3)	52 (12.7)	152 (10.9)	0.306
Unplanned re-operations	128 (7.1)	31 (7.6)	97 (6.9)	0.663

Note: ICD-VR—single chamber implantable cardioverter-defibrillator, ICD-DR—dual chamber implantable cardioverter-defibrillator, S-ICD—subcutaneous implantable cardioverter-defibrillator, CRT-D—cardiac resynchronization therapy defibrillator, ICD—implantable cardioverter-defibrillator.

**Table 2:** Quality of life aspects according to cohorts.

N (%)	Whole Cohort 1809 (100)	Poland 410 (22.7)	Other Countries 1399 (77.3)	*p*-Value
Quality of life after ICD implantation				0.041
Improved	829 (45.8)	209 (51.0)	620 (44.3)	
Unchanged	675 (37.3)	135 (32.9)	540 (38.6)	
Worsened	183 (10.1)	45 (11.0)	138 (9.9)	
I am not sure	122 (6.7)	21 (5.1)	101 (7.2)	
Feeling of safety with an ICD	1302 (80.3)	299 (80.8)	1003 (80.2)	0.787
Feeling of global depression since ICD implantation				0.837
Not at all	928 (57.2)	207 (55.9)	721 (57.6)	
Slightly	362 (22.3)	88 (23.8)	274 (21.9)	
Moderately	235 (14.5)	51 (13.8)	184 (14.7)	
Very	66 (4.1)	15 (4.1)	51 (4.1)	
Extremely	31 (1.9)	9 (2.4)	22 (1.8)	
Acceptance of ICD limitations	1119 (69.2)	255 (68.9)	864 (69.3)	0.893
Significant lifestyle change after ICD implantation	563 (34.8)	126 (34.1)	437 (35.1)	0.718

## Data Availability

The data underlying this article will be shared on reasonable request to the corresponding author with permission of the EHRA Scientific Initiatives Committee.

## Supplementary Materials

The following supporting information can be downloaded at: https://www.mdpi.com/article/10.3390/ijerph20065045/s1, Supplementary Material S1: Questionnaire.
