# Supplementary material for: Implantable Cardioverter-Defibrillators in Poland Compared with other European Countries from the Patient’s Perspective: Insights from the EHRA Patient Survey
Source: Int J Environ Res Public Health. 2023 Mar 13;20(6):5045. doi: 10.3390/ijerph20065045 (PMC10049532; doi:10.3390/ijerph20065045)
Supplement: Supplementary file 1 [file ijerph-20-05045-s001.zip › ijerph-2142799-supplementary.pdf]

Supplementary file 1: Questionnaire

EHRA Patients Survey "Living with an ICD"

Introduction

Dear Sir or Madam,

You are asked to participate in a patient survey entitled "Living with an ICD (Implantable Cardioverter-Defibrillator)" conducted by the European Heart Rhythm Association Scientific Initiatives Committee. The major aim of this survey is to assess the knowledge and quality of life in the ICD recipients. This survey is expected to establish the current needs of ICD patients in terms of information about the device. Study results should help physicians in providing specific counseling in the ICD population. Results of the study will be published in the *Europace* journal.

- Your participation is anonymous and all information you provide for this study will be treated confidentially.
- We will not disclose your identity to any third party.
- We comply with the European General Data Protection Regulation (GDPR) 2016/679. Any personal data processed in connection with this survey will be treated confidentially and only used by the ESC for the purposes of market research and not for promotion. Survey results will be kept for a maximum of 48 months for analysis and quality control purposes. We take all reasonable care to prevent any unauthorized access to your personal data. We respect your privacy and your right to access, modify, or remove your personal data. At any time, you can ask to know what personal data is being held. If you have any questions about data protection or require further information, please contact our data protection officer (DPO) at dpo@escardio.org.
- You have the right to end your participation in this survey at any time.

1 Please confirm that you have read the above and agree to participate in this survey.

- Yes
- No

I Demographic data and device history

2 How old are you? 0-20 / 21-40 / 41-60 / 61-80 / >81

3 Please indicate your gender: Male / Female

4 Country: - ESC Country drop down list + add other with comment box

5 Highest education level: Primary school / Secondary school / College / University

6 Employment status: Student / Employed / Not employed / Retired

7 Marital status: Married or living with a partner / Single / Widower or widow / Living at home (as a child) / Living alone with children

8 Type of ICD implanted: Implantable cardioverter-defibrillator / cardiac resynchronization therapy/defibrillator / subcutaneous implantable cardioverter-defibrillator / I don't know

9 Years since first implantation: slider with range 0-50

10 How would you rate your quality of life after ICD implantation?
- Improved
- Unchanged
- Worsened
- I am not sure

11 Is your device controlled by remote monitoring system? Yes / No / I don't know

12 Why did you have an ICD implanted? (tick all that apply)
- Cardiac arrest
- Prevention of cardiac arrest
- Fatigue, shortness of breath, heart failure
- I don't know

13 Did you experience subsequent complications related to ICD and/or the lead? (tick all that apply)
- Inappropriate shocks

- Unplanned re-operations
- Malfunctioning lead
- None

14 Have you ever been called for unscheduled visit due to a device-related problem?

Yes / No

15 Please describe your feelings related to ICD shock: (tick only one option)

- It was more painful as I expected
- It was as painful as I expected
- It was not as painful as I expected
- I didn’t notice anything (unconscious…)
- I had no ICD shock

II Life with an ICD

16 How would you describe the treatment discussion before the ICD implantation?

- Available treatment options were fully explained to me
- Available treatment options were somewhat explained to me
- Available treatment options were not explained to me

17 How would you describe your participation in the decision about the ICD implantation?

- I was actively involved in the decision-making about the ICD implantation
- I was somewhat involved in the decision-making about the ICD implantation
- I was not at all involved in the decision-making about the ICD implantation

18 Before ICD implantation, I was informed about: (Yes / No)

- Reason for getting ICD
- How the ICD works
- What I could feel if a shock is delivered
- What to do if the ICD delivers a shock
- Possible ICD and lead complications
- How frequently the battery should be replaced

- What I can do physically with an ICD (sports, work, etc.)
- Wound healing and suture removal
- Psychological support post-implantation
- Driving restrictions
- What to do when travelling with an ICD
- My life expectancy
- The possibility to deactivate ICD in the end of life
- In general, I felt well informed before the ICD implantation

19 What aspects about living with an ICD would you like to learn more about? (tick all that apply)

- None, I have sufficient information
- Reason for getting ICD
- How the ICD works
- What I could feel if a shock is delivered
- If it is possible to have normal sexual activity while living with an ICD
- What can our partner feel if the ICD fires during sexual activity
- What bystanders should do if my ICD shocks me?
- How frequently the battery should be replaced
- What to do if the ICD delivers a shock
- Possible ICD complications and how to avoid them
- Allowed and prohibited forms of physical activity
- Allowed and prohibited types of household appliances
- Psychological support post-implantation
- Driving restrictions
- What to do when travelling with an ICD
- Remote monitoring of ICD
- The possibility to deactivate ICD in the end of life

20 How would you prefer to receive information? (tick all that apply)

- Face-to-face discussions
- Audio content (dedicated radio programs, podcasts)
- Video presentations
- Mobile apps
- Internet content
- Printed material (information leaflets, brochures, themed newspapers)

- Other (which)
- I don’t know

21 To what extent do you fear the following ICD issues? (1 – not at all; 2 – a little; 3 – somewhat; 4- quite a bit; 5 – my greatest fear)

- Sudden, unexpected shock
- Shock delivered in public
- Device infection
- Leads extractions
- Malfunctioning leads abandoned in the body
- Long-term device/electrode dysfunction
- Uncertainty about ICD’s proper function and possibility of dying because of ICD failure
- Limitations in my daily life / day to day activities
- Limitations for my work/career
- Limitations for driving

22 To what extent do you agree with the following statements: (1 – strongly disagree, 2 – disagree, 3 – neither agree nor disagree, 4 – agree, 5 – strongly agree)

- I wish I could have had more time before making the decision about ICD implantation
- I wish I could have known more about an ICD before the procedure
- Before implantation my vision of life with ICD was similar to reality after the procedure
- I feel safer with ICD
- Remote monitoring of my ICD would make me feel safer
- Remote monitoring of my ICD would make my daily life easier because it reduces in-hospital visits
- I accept the limitations of ICD and the necessary lifestyle changes
- ICD has had an influence on the way I dress
- ICD has had a negative influence on my professional career
- I had to change my job after ICD implantation
- ICD has changed my lifestyle significantly
- I feel uncomfortable having an ICD
- Having an ICD makes me feeling disable
- I regret my decision about ICD implantation

23 Do you feel globally depressed since your ICD implantation? (1 – not at all, 2 – slightly, 3 – moderately, 4 – very, 5 – extremely)

24 Have you ever heard about support groups of patients with devices?

- Yes, I belong to a group and I find it helpful
- Yes, I belong to a group, but I don't think it is helpful for me
- I do not belong to such a group, but I am interested in one
- Yes, but I do not belong to such a group, and I am not interested
- No

III ICD deactivation

25 Do you agree: (Yes / No)

- I know that ICD may be deactivated at health deterioration
- I understand why ICD deactivation may be helpful
- I believe all patients should be informed about the possibility of deactivating their ICD
- I want to be involved in the ICD deactivation procedure/decision-making
- I have thought what to do with my ICD in case of terminal illness

26 In your opinion, what is the best time to be informed about the possibility of turning off the ICD?

- Before implantation
- Once my condition is stable
- If my health starts to deteriorate
- Other (please describe)

We would like to thank you for your participation in this survey. The results of this research will help us to understand better the contemporary reality of ICD patients and their needs. These results may be useful in providing medical advices for physicians who take care of ICD patients.
